# Mortality in adult patients with culture-positive and culture-negative meningitis in the Botswana national meningitis survey: a prevalent cohort study

**DOI:** 10.1016/S1473-3099(19)30066-0

**Published:** 2019-07

**Authors:** Mark W Tenforde, Margaret Mokomane, Tshepo B Leeme, Nametso Tlhako, Katlego Tsholo, Tony Chebani, Anya Stephenson, Jemima Hutton, Hannah K Mitchell, Raju KK Patel, William J Hurt, Tlhagiso Pilatwe, Tiny Masupe, Mooketsi Molefi, Brandon L Guthrie, Carey Farquhar, Madisa Mine, Joseph N Jarvis

**Affiliations:** a Division of Allergy and Infectious Diseases, Department of Medicine, University of Washington School of Medicine, Seattle, WA, USA; b Department of Epidemiology, University of Washington School of Public Health, Seattle, WA USA; c Botswana-UPenn Partnership, Gaborone, Botswana; d Botswana National Health Laboratory, Gaborone, Botswana; e Botswana Ministry of Health, Gaborone, Botswana; f Department of Family Medicine and Public Health, University of Botswana, Gaborone, Botswana; g Department of Global Health, University of Washington, Seattle, WA USA; h Division of Infectious Diseases, Department of Medicine, Perelman School of Medicine, University of Pennsylvania, Philadelphia, PA, USA; i Botswana Harvard AIDS Institute Partnership, Gaborone, Botswana; j Department of Clinical Research, Faculty of Infectious and Tropical Diseases, London School of Hygiene and Tropical Medicine, London, UK

## Abstract

**Background:**

CNS infections are a leading cause of HIV-related deaths in sub-Saharan Africa, but causes and outcomes are poorly defined. We aimed to determine mortality and predictors of mortality in adults evaluated for meningitis in Botswana, which has an estimated 23% HIV prevalence among adults.

**Methods:**

In this prevalent cohort study, patient records from 2004–15 were sampled from the Botswana national meningitis survey, a nationwide audit of all cerebrospinal fluid (CSF) laboratory records from patients receiving a lumbar puncture for evaluation of meningitis. Data from all patients with culture-confirmed pneumococcal and tuberculous meningitis, and all patients with culture-negative meningitis with CSF white cell count (WCC) above 20 cells per μL were included in our analyses, in addition to a random selection of patients with culture-negative CSF and CSF WCC of up to 20 cells per μL. We used patient national identification numbers to link CSF laboratory records from the national meningitis survey to patient vital registry and HIV databases. Univariable and multivariable Cox proportional hazards models were used to evaluate clinical and laboratory predictors of mortality.

**Findings:**

We included data from 238 patients with culture-confirmed pneumococcal meningitis, 48 with culture-confirmed tuberculous meningitis, and 2900 with culture-negative CSF (including 1691 with CSF WCC of up to 20 cells per μL and 1209 with CSF WCC above 20 cells per μL). Median age was 37 years (IQR 31–46), 1605 (50%) of 3184 patients were male, 2188 (72%) of 3023 patients with registry linkage had documentation of HIV infection, and median CD4 count was 139 cells per μL (IQR 63–271). 10-week and 1-year mortality was 47% (112 of 238) and 49% (117 of 238) for pneumococcal meningitis, 46% (22 of 48) and 56% (27 of 48) for tuberculous meningitis, and 41% (1181 of 2900) and 49% (1408 of 2900) for culture-negative patients. When the analysis of patients with culture-negative CSF was restricted to those with known HIV infection, WCC (0–20 cells per μL *vs* >20 cells per μL) was not predictive of mortality (average hazard ratio 0·93, 95% CI 0·80–1·09).

**Interpretation:**

Mortality from pneumococcal, tuberculous, and culture-negative meningitis was high in this setting of high HIV prevalence. There is an urgent need for improved access to diagnostics, to better define aetiologies and develop novel diagnostic tools and treatment algorithms.

**Funding:**

National Institutes of Health, President's Emergency Plan for AIDS Relief, National Institute for Health Research.

## Introduction

CNS infections are a leading cause of mortality in sub-Saharan Africa.^1^, ^2^ The emergence of HIV markedly altered the epidemiology of meningitis in the region; cryptococcal meningitis, tuberculous meningitis, and bacterial meningitis caused by *Streptococcus pneumoniae* are the most common confirmed causes in adults in central, east, and, southern Africa.^3^, ^4^, ^5^ Outcomes from cryptococcal meningitis have been relatively well described in clinical trials and observational studies.^6^, ^7^, ^8^, ^9^ However, data regarding long-term outcomes from tuberculous or pneumococcal meningitis in routine-care settings are limited.^5^, ^10^, ^11^

**Research in context**

**Evidence before this study**

CNS infections are a leading cause of HIV-related deaths globally. In regions of high HIV prevalence in sub-Saharan Africa, cryptococcal, tuberculous, and pneumococcal meningitis are the most common confirmed aetiologies of meningitis; cryptococcal meningitis alone causes 15% of all HIV deaths. Although cryptococcal meningitis is easily confirmed with widely available and sensitive diagnostic tests and culture is frequently used, additional diagnostic studies are infrequently available. In settings of high HIV prevalence, most individuals who receive a lumbar puncture with cerebrospinal fluid (CSF) analysis for evaluation of infection have no aetiology identified. We searched PubMed, EMBASE, and PubMed Central for studies published between Jan 1, 1990, and Aug 31, 2018, of short-term and long-term outcomes of culture-positive and culture-negative meningitis in routine clinical care settings of sub-Saharan Africa. Search terms included regional search terms for sub-Saharan Africa and meningitis. In-hospital mortality for culture-negative, lymphocyte-predominant meningitis in two small studies of mostly HIV-infected patients was 13% in one study, and 35% in the other. Our search yielded no published studies describing long-term outcomes of patients evaluated for meningitis without a pathogen identified in settings of high HIV prevalence.

**Added value of this study**

We assessed mortality in patients in Botswana with culture-confirmed tuberculous and pneumococcal meningitis and patients with culture-negative meningitis with CSF white cell count (WCC) elevation (>20 cells per μL), as well as a random sample of culture-negative cases with minimal (WCC 0–2 cells per μL) or mild (WCC 3–20 cells per μL) inflammatory changes. Almost three-quarters of patients had known HIV infection, a majority with advanced disease (CD4 count <200 cells per μL), and half of patients without a confirmed pathogen died within 1 year. In the context of advanced HIV infection, CSF WCC was not a predictor of mortality, and tuberculous meningitis was rarely confirmed by culture. To our knowledge, this is the only large study in sub-Saharan Africa to provide robust estimates of short-term and long-term outcomes of both culture-positive and culture-negative meningitis in routine clinical care settings.

**Implications of all the available evidence**

Our findings highlight an exceedingly high mortality among HIV-infected patients evaluated for CNS infection in Botswana, and a poor ability of health-care providers to discriminate who is most at risk of death through available information such as CSF WCC. These findings support an urgent need to scale up existing diagnostics, particularly available molecular diagnostics for tuberculosis, and highlight a need to prioritise research in development and evaluation of sensitive, affordable, and rapid diagnostic tests to inform better management strategies and reduce mortality.

Furthermore, most patients with suspected meningitis evaluated by lumbar puncture and cerebrospinal fluid (CSF) analysis in resource-limited settings have no pathogen identified through diagnostic studies.^3^, ^4^ Even in studies that have used enhanced molecular diagnostics, up to half of patients still did not have a definitive microbiological diagnosis.^12^, ^13^, ^14^ The aetiology of CNS infections therefore remains poorly understood, management recommendations for this large patient population are lacking, and no data exist on short-term or long-term survival.

Botswana, which has an estimated HIV prevalence of 23% among adults, has an electronic national death registry unique within the region, as well as an electronic national HIV registry used for research and surveillance.^15^ We aimed to determine long-term mortality and predictors of mortality in adults evaluated for meningitis in Botswana.

## Methods

### Study design and participants

In this retrospective prevalent cohort study, we compared outcomes for patients with culture-confirmed tuberculous or pneumococcal meningitis, culture-negative meningitis with abnormal CSF cellular findings, and culture-negative cases with a relatively normal CSF profile, and evaluated clinical and laboratory predictors of mortality up to 1 year.

Patient records from Jan 1, 2004, to Dec 31, 2015, were sampled from the Botswana national meningitis survey, a nationwide audit of all CSF laboratory records from patients receiving a lumbar puncture for evaluation of suspected meningitis.^16^

The Botswana national meningitis survey included both paper CSF records and electronic records from a national electronic medical record, the Integrated Patient Management System (IPMS). IPMS was rolled out in Botswana's public hospital laboratory system in 2004, and by 2014 covered over 72% of patients undergoing CSF analysis (appendix). Paper and electronic data were merged, de-duplicated, and stored in an online database (REDCap).

Standard CSF work-up included glucose concentration, protein concentration, and white cell count (WCC) with or without neutrophil and lymphocyte percentages in those patients with elevated WCC. Microbiological testing included Gram stain, India ink stain, and standard bacterial and fungal cultures on all samples. Cryptococcal antigen (CrAg) latex agglutination testing was available in some laboratories. Tuberculosis culture was done at the request of clinical providers at a central national tuberculosis reference laboratory. The Xpert MTB/RIF assay for tuberculosis was introduced in Botswana in August, 2012, but not used on CSF samples. Details of the standard diagnostic work-up for CSF and meningitis treatment regimens in Botswana are listed in the appendix.

Cases of suspected meningitis were defined as culture-negative if no pathogen was identified on standard microbiological testing—ie, they had a negative bacterial culture and Gram stain, negative work-up for cryptococcal meningitis (including microscopy, CrAg testing, and fungal culture), and any tuberculosis microscopy or cultures were negative. For assessment of culture-negative meningitis outcomes the dataset was restricted to patients with a nine-digit national identification number (known as Omang) in IPMS—a unique identification number assigned to all Botswana citizens who are at least 16 years of age. Omang is recorded when patients are registered in IPMS at the time of first hospital admission, and the identification number is verified at each subsequent admission.

All patient records with CSF WCC above 20 cells per μL were selected for analysis, plus a random selection of 1000 CSF samples with negative cultures and normal CSF WCC (0–2 cells per μL) and 1000 CSF samples with mildly abnormal CSF WCC (3–20 cells per μL). We selected 1000 random samples within both of the CSF WCC strata for pragmatic reasons, based on a data agreement with the Botswana Ministry of Labour and Home Affairs which maintains the death registry. Samples were randomly selected on the basis of a random number sequence generated with Stata (version 13.0, College Station, TX, USA). WCC categories were used because mild pleocytosis, typically below 20 cells per μL, is common in asymptomatic HIV-infected adults, and for consistency with previously published data.^4^ Repeat samples from the same patient were excluded, as were patients subsequently found to have positive microscopy in the absence of positive cultures. Children usually do not have an assigned Omang; therefore, selection of culture-negative patients was restricted to individuals who were at least 16 years of age.

The national identification number was used for deterministic linkage of patient CSF laboratory data to HIV-related data in the national HIV registry. Antiretroviral therapy (ART) was available in Botswana starting in 2002, and HIV prevalence in adults (15–49 years) remained stable (from 25% in 2004 to 23% in 2015). We obtained records of HIV status from the HIV registry; for HIV-positive patients we obtained records of their most recent CD4 counts within 6 months of lumbar puncture and on whether the patient had initiated ART before lumbar puncture evaluation.^17^ The date of ART initiation was defined either as ART documented in the registry before the date of lumbar puncture, or in the absence of documented ART initiation, 90 days before the date of first recorded HIV viral load result. This definition was chosen because ART prescription might be incompletely documented and baseline viral load testing is not done in Botswana but recommended 3 months post-ART initiation.^17^ All-cause mortality within 1 year following initial lumbar puncture was assessed through deterministic linkage by unique identification number to the national electronic death registry. From this registry, a previous study^6^ captured vital status for up to 1 year for 93% of patients treated for cryptococcal meningitis at a Botswana referral hospital from 2012–14, suggesting high-quality capture. In 2014, an estimated 89% of deaths were registered within 1 month.^18^

1-year mortality was also assessed for all cases of culture-confirmed *S pneumoniae* meningitis in IPMS (*S pneumoniae* accounted for most cases of culture-confirmed bacterial meningitis) and all cases of culture-confirmed tuberculous meningitis from 2007 onward. Records of culture-confirmed tuberculous meningitis are stored in Disa, Botswana's National Tuberculosis Reference Laboratory's electronic medical record; IPMS does not store tuberculosis culture results. The records were linked to the death registry by Omang (when available) or by first name and surname, and by Omang only to the HIV registry.

The study was approved by institutional review boards at the University of Pennsylvania (Philadelphia, PA, USA), University of Washington (Seattle, WA, USA), and University of Botswana (Gaborone, Botswana), and by the Health Research and Development Committee of the Botswana Ministry of Health (Gaborone, Botswana). As the study involved only retrospective collection of routine clinical and outcomes data, a waiver of informed patient consent was obtained.

### Outcomes

The main study outcomes included 10-week and 1-year mortality for pneumococcal, tuberculous, and culture-negative meningitis, and clinical and laboratory predictors of mortality.

### Statistical analysis

Demographic, clinical, and laboratory characteristics were described with summary statistics. The χ^2^ and Wilcoxon rank sum tests were used to compare categorical and continuous characteristics between groups. Kaplan-Meier survival curves were generated for tuberculous meningitis and pneumococcal meningitis cases, and for culture-negative cases.

Univariable Cox proportional hazards models were constructed with 10-week and 1-year mortality data from first lumbar puncture as the outcome, to evaluate characteristics associated with short-term and long-term mortality. Because patients without known HIV in this dataset are likely to represent a mix of HIV-uninfected individuals and HIV-infected but undiagnosed individuals, an analysis restricted to HIV-infected patients was done. Variables were dichotomised with clinically meaningful or previously established cutoffs:^4^, ^6^, ^8^ older age (≥50 years), elevated CSF WCC (>20 cells per μL), lymphocyte-predominant CSF (≥50%), low CSF glucose concentration (<1 mmol/L), high CSF protein concentration (≥1 g/dL), and facility type (referral hospital *vs* primary or district hospital). CD4 cell counts were stratified as above 350 cells per μL, 200–350 cells per μL, 100–200 cells per μL, 50–100 cells per μL, and up to 50 cells per μL. We also assessed hazards of death by calendar year to assess temporal trends.

Multivariable Cox proportional hazards models were constructed with older age, sex, CSF WCC above 20 cells per μL, CD4 strata, ART status, facility level, and calendar year as independent variables. Variables were selected a priori because of suspected causal association with mortality to evaluate independent predictors of death. A subgroup analysis restricted to HIV-infected patients with CSF WCC above 20 cells per μL was done to evaluate additional associations between CSF profiles (lymphocyte predominance *vs* neutrophil predominance) and mortality. CSF chemistries (protein and glucose concentrations) and HIV viral loads were excluded from multivariable models because of high degree of missingness, and in the case of viral load, a high likelihood that data were not missing at random. Because of the fewer cases of culture-confirmed pneumococcal meningitis, only univariable regression analyses were done and restricted to 10-week mortality, because most deaths occurred within the first 10 weeks after lumbar puncture. Univariable models estimated mortality hazard ratios per 100 cells per μL difference in WCC, 1 g/L difference in CSF protein concentration, and 1 mmol/L difference in CSF glucose concentration. p values were obtained with conventional standard errors (significance level p≤0·05). Statistical analyses were done in Stata (version 13·0, College Station, TX, USA).

### Role of the funding source

The funders for this study had no role in study design, data collection, data analysis, data interpretation, writing of this report, or decision to submit this paper for publication. MWT had full access to all the data in the study and had final responsibility for the decision to submit for publication.

## Results

The Botswana national meningitis survey included 29 704 CSF records from 2004–15, representing 27 432 unique cases after repeat CSF samples from a single admission were excluded. Among all CSF records from 2004–15, 21 941 (80%) of 27 432 had no pathogen identified and 5491 (20%) had one or more pathogens identified. Cryptococcal meningitis accounted for 4532 (82%) of 5491 microbiological diagnoses, tuberculous meningitis for 63 (1%), and pneumococcal or other bacterial meningitis for 977 (18%), with 45 (1%) having multiple diagnoses.

**Figure 1:**
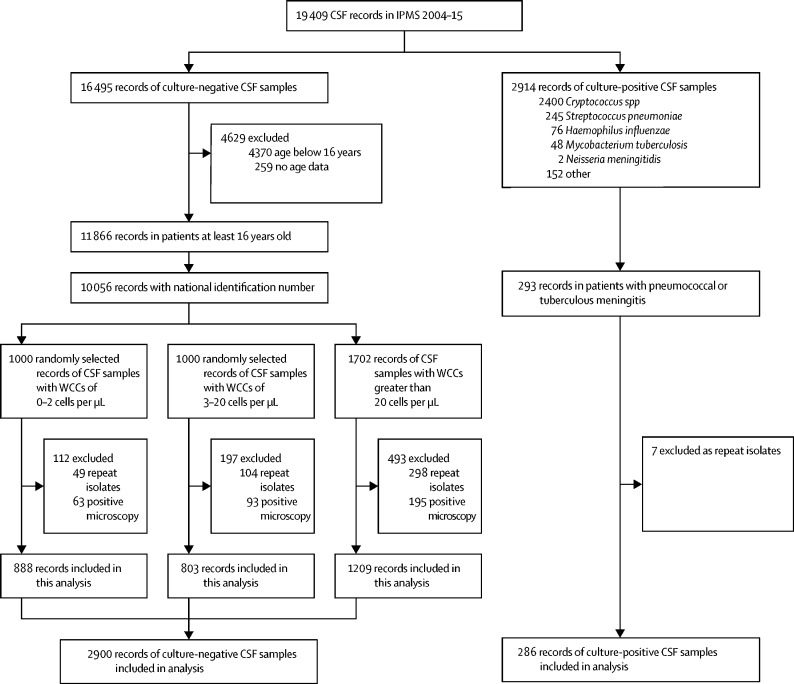
Sampling method for selection of patients with culture-positive and culture-negative CSF IPMS=Integrated Patient Management System. WCC=white cell count.

19 409 (65%) of the 29 704 CSF records included in the Botswana national meningitis survey were in IPMS and sampled for this analysis. We included data from 3186 patients in our analysis: 238 with culture-confirmed pneumococcal meningitis, 48 with culture-confirmed tuberculous meningitis, and 2900 with culture-negative CSF (including 1691 with CSF WCC of up to 20 cells per μL and 1209 with CSF WCC greater than 20 cells per μL; table 1). Although India ink and fungal cultures are routinely done, CrAg testing was done on only 703 (4%) of 19 409 samples registered on IPMS during the study period. Tuberculous meningitis testing was rare, with culture done on 1723 (9%) of 19 409 samples and AFB microscopy on 2524 (13%). Tuberculosis cultures were obtained from Disa; 39 of those 48 patients also had a record in the IPMS. The cohorts of patients with culture-positive or culture-negative meningitis are described in figure 1. Overall, the median age of patients sampled in the study was 37 years (IQR 31–46); 1605 (50%) of 3184 patients were male, 2188 (72%) of 3023 patients with registry linkage had documentation of HIV infection, and median CD4 count was 139 cells per μL (IQR 63–271). 2345 (74%) of 3186 patients were treated at one of two national referral hospitals. For patients with culture-negative meningitis, 2109 (73%) of 2900 had documented HIV infection. CD4 cell count was measured within 6 months of lumbar puncture in 1578 (75%) of these 2109 HIV-positive individuals, with a median 136 cells per μL (IQR 61–266). 956 (45%) of 2109 patients with known HIV were on ART by the date of lumbar puncture; 615 (64%) of these 956 had a documented ART start date in IPMS, and 341 (36%) were classified as being on ART based on previous viral load testing. Compared with the 1691 patients with low CSF WCC (≤20 cells per μL), the 1209 patients with elevated CSF WCC (>20 cells per μL) were more likely to be male (661 [55%] patients with elevated CSF WCC *vs* 823 [49%] patients with low CSF WCC; p=0·001) and less likely to be on ART before lumbar puncture (390 [43%] patients with elevated CSF WCC *vs* 605 [50%] patients with low CSF WCC; p=0·003), but had a marginally higher median CD4 count (median 141 cells per μL [IQR 73–270] in patients with elevated CSF WCC *vs* 133 cells per μL in patients with low CSF WCC [51–265]; p=0·010). Previous known HIV status did not differ significantly between CSF WCC strata (898 [74%] in patients with elevated CSF WCC *vs* 1221 [72%] in patients with low CSF WCC; p=0·11).

**Table 1:** Baseline clinical and laboratory characteristics and mortality outcomes

		**Culture-confirmed bacterial meningitis**		**No pathogen identified**	
		Pneumococcal meningitis (n=238)	Tuberculous meningitis (n=48)	CSF white cell count 0–20 cells per μL (n=1691)	CSF white cell count above 20 cells per μL (n=1209)
Age (years)		32·6 (17·7–42·3)	34·1 (23·8–45·2)	37·8 (31·2–46·8)	37·0 (30·9–44·7)
Sex					
	Female	141 (59%)	24 (50%)	868 (51%)	548 (45%)
	Male	97 (41%)	24 (46%)	823 (49%)	661 (55%)
	Unknown	··	2 (4%)	*··*	*··*
CSF white cell count (cells per μL)*		236 (53–800)	65 (25–185)	2 (2–5)	115 (50–325)
Lymphocytes (%)		10% (5–25)	93% (32–97)	90% (70–98)	88% (42–95)
Protein (g/dL)		4·14 (2·5–7·1)	3·13 (2·6–3·5)	0·55 (0·30–1·07)	1·89 (1·03–3·42)
Glucose (mmol/L)		0·04 (0·01–0·12)	2·73 (1·99–3·48)	3·22 (2·57–3·83)	1·85 (1·07–2·84)
HIV positive		79 (64%)	22 (76%)	1211 (72%)	898 (74%)
If HIV positive					
	CD4 T-cell count (cells per μL)	221 (141–421)	105 (34–162)	133 (51–265)	141 (73–270)
	On antiretroviral therapy	35 (44%)	··	605 (50%)	390 (43%)
2-week mortality (%)		44·1% (105)	37·5% (18)	28·6% (484)	29·4% (355)
10-week mortality (%)		47·1% (112)	45·8% (22)	40·1% (678)	41·6% (503)
1-year mortality (%)		49·2% (117)	56·3% (27)	48·4% (819)	48·7% (589)

Data are median (IQR) or n (%), or % (n). CSF=cerebrospinal fluid.

* The upper limit recorded for white cell count was 2000 cells per μL.

999 (83%) of 1209 patients with culture-negative CSF and elevated CSF WCC (WCC >20 cells per μL) had differential cell counts; 734 (73%) of 999 had lymphocyte-predominant CSF. Only 78 of 1691 patients with low CSF WCC (≤20 cells per μL) had differential counts; 64 (82%) of 78 had lymphocyte-predominant CSF. CSF protein concentration was higher and glucose concentration lower in patients with CSF WCC above 20 cells per μL, compared with those who had CSF WCC of up to 20 cells per μL (all p<0·0001).

**Figure 2:**
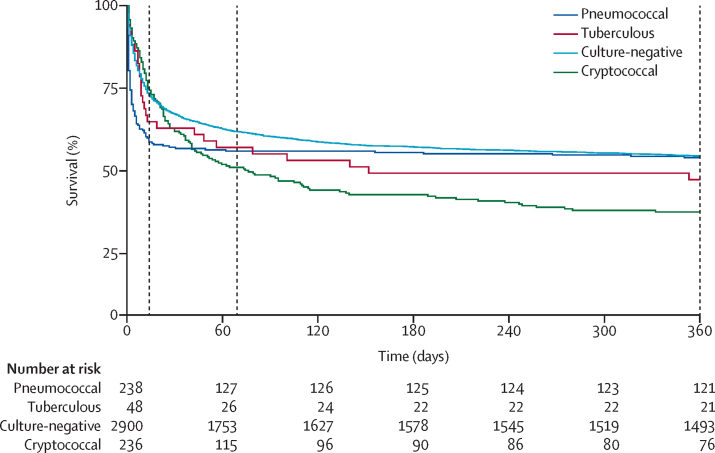
Kaplan-Meier survival analysis of patients with pneumococcal meningitis, tuberculous meningitis, culture-negative meningitis, and cryptococcal meningitis Dashed vertical lines represent 2 weeks, 10 weeks, and 1 year. Cryptococcal meningitis data are previously published 2012–2014 referral hospital outcomes for comparison.^6^

**Figure 3:**
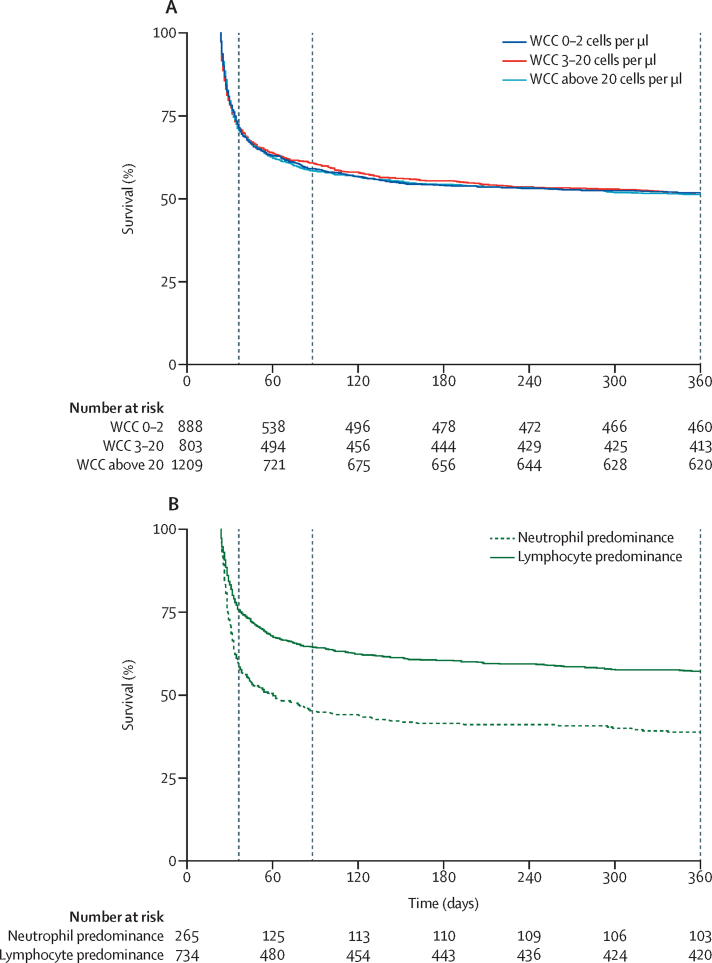
Kaplan-Meier analysis of survival for patients with culture-negative meningitis (A) Probability of survival of patients with CSF WCC 0–2 cells per μL, CSF WCC 3–20 cells per μL, and CSF WCC above 20 cells per μL. (B) Probability of survival of patients with CSF WCC above 20 cells per μL by neutrophil versus lymphocyte (≥50% lymphocytes) predominance. Dashed vertical lines represent 2 weeks, 10 weeks, and 1 year. Note that a proportion of patients with WCC above 20 cells per μL did not have a WCC differential. CSF=cerebrospinal fluid. WCC=white cell count.

Kaplan-Meier survival curves are shown in figure 2 for patients with pneumococcal meningitis, tuberculous meningitis, and culture-negative meningitis, as well as previously published 2012–14 referral hospital outcomes from cryptococcal meningitis for comparison.^6^ For culture-confirmed pneumococcal meningitis, 2-week mortality was 44% (105 of 238), 10-week mortality was 47% (112 of 238), and 1-year mortality was 49% (117 of 238; table 1). 105 (90%) of those 117 deaths occurred within 2 weeks of CSF evaluation. For culture-confirmed tuberculous meningitis, 2-week mortality was 38% (18 of 48), 10-week mortality was 46% (22 of 48), and 1-year mortality 56% (27 of 48). For culture-negative patients, overall 2-week mortality was 29% (839 of 2900), 10-week mortality was 41% (1181 of 2900), and 1-year mortality was 49% (1408 of 2900). There was no significant difference in risk of mortality between WCC strata (p>0·05 for 2-week, 10-week, and 1-year mortality; figure 3). In both the pneumococcal meningitis cohort and the culture-negative cohort, mortality was higher in patients without known HIV diagnosis. 44 (36%) of 123 patients with culture-confirmed pneumococcal meningitis and HIV registry linkage had unknown HIV status; for those patients, 2-week mortality was 77% (34 of 44), 10-week mortality was 82% (36 of 44), and 1-year mortality was 82% (36 of 44). 791 (27%) of 2900 patients with culture-negative CSF and HIV registry linkage had unknown HIV status; for those patients, 2-week mortality was 43% (344 of 791), 10-week mortality was 55% (438 of 791), and 1-year mortality was 62% (487 of 791).

For patients with culture-confirmed pneumococcal meningitis, univariable analysis showed that increased CSF WCC was associated with lower risk of mortality within 10 weeks of lumbar puncture (hazard ratio [HR] 0·96, 95% CI 0·92–0·99 for each increase in WCC of 100 cells per μL); when the analysis was restricted to those with known HIV infection, increased WCC was also associated with lower risk of mortality (HR 0·93, 95% CI 0·87–0·99 for each increase in WCC of 100 cells per μL; appendix). However, higher CSF protein concentration in these patients was associated with higher risk of mortality (HR 1·04, 95% CI 1·00–1·09 for 1 g/L increase in protein; appendix).

Univariable analysis in HIV-infected patients with negative cultures showed that older age, male sex, low CSF glucose concentration, lower CD4 cell count, and treatment at non-tertiary facilities were significantly associated with increased risk of mortality within 1 year (table 2). Calendar year was not associated with risk of death (data not shown). Adjusting for other covariates in multivariable analyses, older age and lower CD4 cell count were associated with higher risk of mortality, and baseline ART use with lower risk of mortality (table 3).

**Table 2:** Univariable analysis of mortality in HIV-infected patients without a pathogen identified

	**N (%)**	**10-week mortality**			**1-year mortality**		
		**n/N (%)**	**HR (95% CI)**	**p value**	**n/N (%)**	**HR (95% CI)**	**p value**
**Age (n=2109)**							
Below 50 years	1830 (87%)	598/1830 (33%)	1 (ref)	p<0·0001	752/1830 (41%)	1 (ref)	p<0·0001
At least 50 years	279 (13%)	145/279 (52%)	1·80 (1·50–2·16)	··	169/279 (61%)	1·75 (1·48–2·06)	··
**Sex (n=2109)**							
Female	1069 (51%)	345/1069 (32%)	1 (ref)	p=0·0059	425/1069 (40%)	1 (ref)	p=0·0006
Male	1040 (49%)	398/1040 (38%)	1·22 (1·06–1·41)	··	496/1040 (48%)	1·26 (1·10–1·43)	··
**WCC category (n=2109)**							
0–20 cells per μL	1211 (57%)	431/1211 (36%)	1 (ref)	p=0·53	539/1211 (45%)	1 (ref)	p=0·306
Above 20 cells per μL	898 (43%)	312/898 (35%)	0·95 (0·83–1·10)	··	382/898 (43%)	0·93 (0·82–1·06)	··
**CSF protein concentration (n=673)**							
Below 1 g/L	350 (52%)	102/350 (29%)	1 (ref)	p=0·0078	136/350 (39%)	1 (ref)	p=0·026
At least 1 g/L	323 (48%)	126/323 (39%)	1·43 (1·10–1·85)	··	151/323 (47%)	1·30 (1·03–1·64)	··
**CSF glucose concentration (n=1031)**							
At least 1 mmol/L	907 (88%)	318/907 (35%)	1 (ref)	p=0·051	394/907 (43%)	1 (ref)	p=0·013
Below 1 mmol/L	124 (12%)	55/124 (44%)	1·33 (1·00–1·77)	··	69/124 (56%)	1·38 (1·07–1·78)	··
**CD4 strata (n=1578)**							
Above 350 cells per μL	261 (17%)	54/261 (21%)	1 (ref)	p<0·0001	73/261 (28%)	1 (ref)	p<0·0001
200–350 cells per μL	299 (19%)	87/299 (29%)	1·48 (1·05–2·07)	··	112/299 (38%)	1·43 (1·07–1·92)	··
100–200 cells per μL	388 (25%)	131/388 (34%)	1·76 (1·28–2·42)	··	173/388 (45%)	1·78 (1·35–2·33)	··
50–100 cells per μL	310 (20%)	144/310 (46%)	2·61 (1·91–3·57)	··	175/310 (56%)	2·52 (1·92–3·31)	··
At least 50 cells per μL	320 (20%)	159/320 (50%)	2·82 (2·07–3·84)	··	189/320 (59%)	2·67 (2·04–3·50)	··
**ART status (n=2109)**							
Off ART	1114 (53%)	409/1114 (37%)	1 (ref)	p=0·18	498/1114 (45%)	1 (ref)	p=0·32
On ART	995 (47%)	334/995 (34%)	0·91 (0·78–1·05)	··	423/995 (43%)	0·94 (0·82–1·07)	··
**Facility type (n=2109)**							
Primary or district hospital	582 (28%)	235/582 (40%)	1 (ref)	p=0·0017	287/582 (49%)	1 (ref)	p=0·0007
Referral hospital	1527 (72%)	508/1527 (33%)	0·78 (0·67–0·91)	··	634/1527 (42%)	0·79 (0·68–0·90)	··

CSF=cerebrospinal fluid. HR=hazard ratio

**Table 3:** Multivariable analysis of mortality in HIV-infected patients without a pathogen identified

	**10-week mortality**		**1-year mortality**	
	**aHR (95% CI)**	**p value**	**aHR (95% CI)**	**p value**
**Age**				
<50 years	1 (ref)	p<0·0001	1 (ref)	p<0·0001
≥50 years	1·74 (1·41–2·14)	··	1·65 (1·36–2·00)	··
**Sex**				
Female	1 (ref)	p=0·73	1 (ref)	p=0·68
Male	1·02 (0·87–1·22)	··	1·03 (0·89–1·20)	··
**WCC category**				
0–20 cells per μL	1 (ref)	p=0·68	1 (ref)	p=0·38
Above 20 cells per μL	0·96 (0·81–1·14)	··	0·93 (0·80–1·09)	··
**CD4 strata**				
>350 cells per μL	1 (ref)	p<0·0001	1 (ref)	p<0·0001
200–350 cells per μL	1·51 (1·08–2·13)	··	1·47 (1·10–1·98)	··
100–200 cells per μL	1·80 (1·31–2·48)	··	1·83 (1·39–2·42)	··
50–100 cells per μL	2·56 (1·87–3·52)	··	2·50 (1·90–3·30)	··
≤50 cells per μL	2·85 (2·08–3·90)	··	2·71 (2·06–3·57)	··
**ART status**				
Off ART	1 (ref)	p<0·0001	1 (ref)	p<0·0001
On ART	0·70 (0·59–0·83)	··	0·74 (0·64–0·87)	··
**Facility type**				
Primary or district hospital	1 (ref)	p=0·062	1 (ref)	p=0·068
Referral hospital	0·84 (0·70–1·01)	··	0·86 (0·73–1·01)	··

Analyses were adjusted for age, sex, WCC category, CD4 strata, ART status, year, and facility level. aHR=average hazard ratio. WCC=white cell count. ART=antiretroviral therapy.

In an adjusted analysis restricted to HIV-infected patients with clear evidence of CSF inflammation (WCC >20 cells per μL), lymphocyte-predominant CSF was associated with lower mortality at 10 weeks and 1 year after lumbar puncture (appendix). Mortality at 2 weeks, 10 weeks, and 1 year was 42% (110 of 265), 55% (145 of 265), and 61% (162 of 265) for patients with neutrophil predominance and CSF WCC above 20 cells per μL; and 24% (179 of 734), 36% (261 of 734), and 43% (314 of 734) for patients with lymphocyte predominance. Older age and lower CD4 cell count were associated with a significantly increased risk of death within 10 weeks and 1 year of lumbar puncture, and ART use at the time of lumbar puncture with lower risk of death (appendix).

## Discussion

In this routine-care setting with high HIV prevalence in sub-Saharan Africa, almost half of the 3186 patients we evaluated for CNS infection died within 1 year of lumbar puncture. These figures represent conservative mortality estimates, as a small proportion of deaths in Botswana probably go unregistered.^18^ Three-quarters of patients had known HIV infection with a median CD4 count below 150 cells per μL; however, the high mortality seen in these patients cannot be attributed to advanced HIV infection alone. For comparison, in 2002–13 the estimated 1-year mortality in patients (with a median baseline CD4 count of 147 cells per μL) newly accessing ART in the Botswana ART programme was 4·3%.^15^ Mortality was high in both culture-confirmed tuberculous and pneumococcal meningitis, with very rapid mortality in patients with pneumococcal meningitis. Our data showed similarly high mortality in patients with culture-negative meningitis, without a pathogen identified through available microbiological studies. Mortality did not differ in patients with clear evidence of CSF inflammation and in those with relatively normal CSF cell count, although neutrophil-predominant CSF predicted poor outcome in patients with pleocytosis. To our knowledge, with data from over 3000 unique patients, this is the first large study to describe long-term mortality of patients evaluated for CNS infections in a setting of high HIV prevalence.

Diagnosis of CNS infections is challenging in sub-Saharan Africa, owing to limited laboratory and imaging infrastructure and the effect of HIV infection on CSF findings.^4^ A substantial majority of individuals with meningitis in routine-care settings in Botswana had a negative diagnostic evaluation, similar to previous studies from other countries in sub-Saharan Africa with high HIV prevalence.^3^, ^4^ Undiagnosed cryptococcal meningitis may have accounted for some cases of culture-negative meningitis. CrAg testing was infrequently done and the highly sensitive IMMY lateral flow assay (Norman, OK, USA) was not used in Botswana during the study period;^19^ however, standard evaluation with combination India ink microscopy and fungal culture is likely to detect most cases. Bacterial meningitis cases may be under-diagnosed with conventional culture-based methods; preliminary findings from a molecular diagnostics study at Botswana's largest referral hospital showed that six (38%) of 16 patients with pneumococcal meningitis were diagnosed by PCR alone, with negative culture results (Jarvis JN, unpublished). Cases may represent undiagnosed tuberculosis, other serious opportunistic infections, or viral pathogens that require molecular diagnostics for confirmation; our findings of extremely high mortality within 1 year of evaluation strongly suggest severe underlying pathology in many patients, requiring improved diagnostics to enable effective treatment.

Undiagnosed tuberculous meningitis may have contributed to the high mortality in culture-negative cases, even in those patients with relatively normal CSF cell count. Compared with HIV-uninfected patients with tuberculous meningitis, patients with advanced HIV are more likely to have atypical CSF findings including neutrophil predominance or relatively acellular CSF.^20^ Culture-confirmed tuberculous meningitis was uncommon in Botswana compared with previous studies in South Africa, where CSF testing for tuberculous meningitis is more common.^4^, ^5^ In 2017, estimated overall tuberculosis incidence was 300 cases per 100 000 person-years in Botswana and 567 cases per 100 000 person-years in South Africa.^21^ Xpert MTB/RIF, endorsed by WHO for the diagnosis of tuberculous meningitis,^22^ has limited sensitivity and was not used in Botswana during the study period, and tuberculosis culture has a suboptimal sensitivity of around 50–60%.^23^ Second-generation Xpert MTB/RIF (Ultra) has improved sensitivity over culture and first-generation Xpert MTB/RIF in HIV-infected patients,^24^ making this an attractive diagnostic option. Our findings strongly suggest a need for an enhanced diagnostic approach in settings with high HIV prevalence and tuberculosis burden, including evaluation for tuberculous meningitis for all cases of suspected meningitis without another clear underlying aetiology. Evidence of tuberculosis elsewhere in the body, coupled with CSF and imaging findings, may also suggest probable tuberculous meningitis and guide treatment.^23^

Molecular diagnostic studies in African health settings suggest that HIV-associated CNS infections due to treatable infections, including herpes viruses and *Toxoplasma gondii*, are common and under-diagnosed in routine care when enhanced diagnostics are not available.^12^, ^13^, ^14^ Development and scale-up of sensitive and affordable multiplex assays for meningitis may assist in guiding therapy and improving outcomes. Further detailed studies to define underlying aetiologies are needed to inform how best to develop and deploy such diagnostics.

We found significantly higher mortality in patients evaluated for meningitis without known HIV infection, almost certainly reflecting a survival bias in which patients with undiagnosed advanced HIV were dying shortly after hospital presentation, before a HIV diagnosis could be established. Although almost three-quarters of patients evaluated for meningitis without a pathogen identified and 64% of those with pneumococcal meningitis were known to be HIV-infected, these figures likely underestimate true HIV prevalence. For example, HIV prevalence in previous studies in Malawi and South Africa was at least 90% in patients with bacterial meningitis.^4^, ^25^ In our study, CD4 cell count was an important predictor of mortality in patients with known HIV infection, similar to previous analyses.^26^ ART use before evaluation was also associated with improved survival, likely from a combination of improved outcomes for certain CNS infections (eg, progressive multifocal leukoencephalopathy) and decreased risk death from other HIV-related co-infections or malignancies. In patients with confirmed pneumococcal meningitis, we found that lower CSF leucocyte counts were significantly associated with higher mortality, as has been previously described in HIV-associated cryptococcal and tuberculous meningitis.^8^, ^27^ Our findings suggest that absence of a robust immune response might be an important predictor of mortality in HIV-related bacterial meningitis, in part, explaining why clinical trials have not shown a mortality benefit with adjunctive glucocorticoid therapy in HIV-infected patients.^25^

Neutrophil-predominant meningitis was associated with higher mortality than lymphocyte-predominant meningitis. This could be in part attributable to bacterial meningitis being common among these neutrophil-predominant culture-negatives cases, with negative culture results caused by previous antibiotic exposure, lumbar puncture delays, or inadequate culture methods. However, overall mortality in patients with lymphocytic meningitis was still high at 36% at 10 weeks and 43% at 1 year. This study improves our understanding of outcomes of culture-negative lymphocytic meningitis. One small study in the Central African Republic^28^ found a 13% (6/46) in-hospital mortality for HIV-infected patients admitted with lymphocytic meningitis (defined as CSF WCC >5 cells per μL, >50% mononuclear cells, and negative bacterial and fungal microscopy and culture), whereas a study from Zimbabwe^11^ found a 35% (15/43) in-hospital mortality for HIV-infected patients with lymphocytic meningitis (defined as CSF WCC >100 cells per μL with >50% mononuclear cells). Our findings highlight how lymphocytic meningitis cannot be discounted as a benign viral or other self-limited process in settings of high HIV prevalence.

Our study has several limitations. First, detailed information on patients' clinical presentation, evaluations including imaging studies, and clinical management including prior antimicrobial use were not available. Second, HIV-related data were derived from an electronic registry that is incomplete, particularly with respect to ART status and history including treatment default.^15^ Third, there was substantial missingness of data for some variables and likely non-random missingness (eg, for HIV viral load), which precluded a multiple imputation approach to missing data and inclusion of certain variables in adjusted regression models. Fourth, we restricted our analysis to a sample of adults in the electronic medical record with available national identification numbers, potentially missing a small proportion of non-citizens and over-representing patients receiving care at referral centres. We believe that our sampling method resulted in conservative mortality estimates compared with the full cohort of adults receiving evaluation for meningitis, as non-citizens are likely to have worse access to HIV and other health services, and patients cared for at referral centres had lower mortality than patients at primary or district hospitals in Botswana. Fifth, in any prevalent cohort study the possibility of lead-time bias exists. However, most patients with meningitis present to health facilities for diagnostic evaluation rapidly on becoming acutely symptomatic, thus lead-time bias is unlikely to occur. Finally, it was necessary to make certain assumptions that might have led to misclassification of a small number of patients (eg, in determining ART start date). We also assumed that all patients received a lumbar puncture for suspected CNS infection. This assumption is supported by data from a prospective cohort study at Botswana's main referral hospital where lumbar punctures done between January, 2017, and June, 2018, were almost exclusively for evaluation of suspected CNS infection rather than for evaluation of cancer, autoimmune disorders, or other non-infectious work-up (Jarvis JN, unpublished).

In summary, we found very high mortality for cases of pneumococcal and tuberculous meningitis, indicating the need for earlier diagnosis and better therapies for patients treated in HIV-prevalent, resource-limited settings. The large majority of meningitis cases were in patients with culture-negative CSF and unconfirmed diagnoses, and these patients had a similarly high mortality up to 1 year, suggesting serious undiagnosed pathology. Our ability to stratify mortality risk by CSF characteristics alone (particularly by CSF WCC) was limited, and our findings highlight an urgent need for improved access to diagnostics (eg, Xpert and multiplex PCR assays) to better define aetiologies and develop novel diagnostic tools and treatment algorithms.

For more on **Botswana HIV and AIDS estimates** see http://www.unaids.org/en/regionscountries/countries/Botswana
