# Supplementary material for: Mortality in adult patients with culture-positive and culture-negative meningitis in the Botswana national meningitis survey: a prevalent cohort study
Source: Lancet Infect Dis. 2019 Jul;19(7):740–9. doi: 10.1016/S1473-3099(19)30066-0 (PMC7645732; doi:10.1016/S1473-3099(19)30066-0)
Supplement: Supplementary appendix [file mmc1.pdf]

THE LANCET
Infectious Diseases

Supplementary appendix

This appendix formed part of the original submission and has been peer reviewed. We post it as supplied by the authors.

Supplement to: Tenforde MW, Mokomane M, Leeme TB, et al. Mortality in adult patients with culture-positive and culture-negative meningitis in the Botswana national meningitis survey: a prevalent cohort study. *Lancet Infect Dis* 2019; **19:** 740–49.

Supplementary Table 1. Microbiological testing, treatment, and record keeping for suspected cases of meningitis in Botswana

**1. Diagnostics**						
a) Laboratory Standard Procedure for Processing Cerebrospinal Fluid (CSF) – all samples						
Macroscopic examination; total cell count using Neubauer counter; centrifugation of CSF at 3000 revolutions per minute for 3 minutes followed by Gram stain, India ink stain, differential count (if CSF white cell count $\geq 10/mm^3$) and culture using the sediment on (i) sabouraud dextrose agar, (incubation 10 days), (ii) sheep blood agar (incubation 72 hours), and (iii) chocolate agar (incubation 72 hours).						
b) Laboratory Standard Procedure for Processing Cerebrospinal Fluid (CSF) – on request						
Acid Fast Bacilli smear: performed on 13% (2,524/19,409) of samples registered in the Integrated Patient Management System (IPMS) during the study period.<br>TB culture on Lowenstein-Jensen slopes from 2004 to 2011, then Bactec 960 Mycobacterium Growth Indicator Tube (MGIT) automated culture system: performed on 9% (1,723/19,409) of samples registered in the Integrated Patient Management System (IPMS) during the study period.<br>Cryptococcal antigen testing (latex agglutination, various manufacturers): performed on 4% (703/19,409) of samples registered in the Integrated Patient Management System (IPMS) during the study period.						
**2. Treatment**						
Cryptococcal meningitis: 2004-2012: induction amphotericin B (0.7-1 mg/kg/day) intravenously (IV) for 2 weeks, then consolidation with fluconazole 400 mg/day orally (PO)/IV for 8-10 weeks; 2012 guidelines: induction amphotericin B (0.7-1 mg/kg/day) IV and fluconazole 800 mg/day PO/IV for 2 weeks, then consolidation with fluconazole 800 mg/day PO/IV for 8 weeks.						
TB meningitis: 2 months HRZE followed by at least 6 months HR with adjunctive steroids for patients with TB meningitis with decreased consciousness or neurological deficits as Prednisolone 60 mg per day for 4 weeks then tapered over several weeks.						
Bacterial meningitis: ceftriaxone 2 grams IV every 12 hours.						
Other: Toxoplasmosis encephalitis: cotrimoxazole 15 mg/kg PO/IV every 8 hours for 6 weeks (first-line with pyrimethamine plus clindamycin generally unavailable); HSV meningoencephalitis: acyclovir 10 mg/kg IV every 8 hours for 21 days; Disseminated VZV: acyclovir 10 mg/kg IV every 8 hours for 7-10 days; CMV encephalitis: no specific recommendations in treatment guidelines, but typical treatment is with ganciclovir/valganciclovir (intermittently available in Botswana).						
**3. Electronic Medical Records**						
Year*	2010	2011	2012	2013	2014	2015
Proportion of CSF samples processed in IPMS facility	34% (974/2858)	45% (1325/2932)	64% (1846/2884)	66% (1892/2874)	72% (2138/2971)	>80% †

HRZE = isoniazid, rifampin, pyrazinamide, ethambutol; HR = isoniazid, rifampin; IPMS = Integrated Patient Management System.

*Prior to 2010 there was incomplete coverage of paper records, thus an accurate estimate of the proportion of samples processed in IPMS facilities is not possible, however it is likely to have remained static at around 30% as there was limited expansion of IPMS coverage during this time, and the number of IPMS samples processed remained relatively constant: 1527 in 2004, 1720 in 2005, 1801 in 2006, 1804 in 2007, 1349 in 2008, 1332 in 2009.
†All laboratories processing CSF in Botswana transitioned to the Integrated Patient Management System in 2015 with the exception of two private laboratories and one public sector facility accounting for 7.42% of all samples processed in 2014. Paper data collection discontinued in 2015 due to this transition so exact figures are unavailable but assuming the proportional workload remained the same we estimate approximately 92% coverage.

Supplementary Table 2. Diagnostic classification of patients included in the Botswana National Meningitis Survey (2004-2015)

**Diagnostic classification**	**All Records*** **(n=27,432)**	**IPMS Records (n=17,514)**	**Paper Records (n=9,918)**
No microbiological diagnosis	***21,941 (80.0%)***	***13,964 (79.7%)***	***7,977 (80.4%)***
CSF WCC<5/µL (or not reported†)	17245 (62.9%)	10809 (61.7%)	6436 (64.9%)
CSF WCC 6-20/µL	1765 (6.4%)	1148 (6.6%)	617 (6.2%)
CSF WCC >20/µL	2951 (10.8%)	2007 (11.5%)	944 (9.3%)
No differential	*571*	*260*	*311*
Lymphocyte predominant§	*1589 (67%)*	*1218 (70%)*	*371 (61%)*
Neutrophil predominant§	*771 (33%)*	*529 (30%)*	*242 (39%)*
Microbiological diagnosis¶	***5,491 (20.0%)***	***3,550 (20.3%)***	***1,941 (19.6%)***
Cryptococcal Meningitis	4532 (16.5%)	2962 (16.7%)	1570 (15.8%)
Tuberculous Meningitis	63 (0.2%)	35 (0.2%)	28 (0.3%)
Bacterial Meningitis	977 (3.6%)	607 (3.5%)	370 (3.7%)
S. pneumoniae	*537(55%)*	*325 (54%)*	*212 (57%)*
H. influenzae	*121 (12%)*	*76 (13%)*	*45 (12%)*
N. meningitidis	*24 (2%)*	*12 (2%)*	*12 (3%)*
*Other***	*295 (30%)*	*194 (32%)*	*101 (27%)*

IPMS = Integrated Patient Management System, the Botswana electronic medical records system. CSF = cerebrospinal fluid. WCC = white cell count.
* The Botswana National Meningitis Survey included 29,704 cerebrospinal fluid (CSF) samples from 27,432 distinct clinical episodes. The presented figures are deduplicated by clinical episode. Therefore the 19,409 IPMS samples represented 17,514 clinical episodes after accounting for multiple lumbar punctures during a single admission.
† CSF white cell counts were not reported in 2,111 IPMS records (12%) and 2,440 paper records (25%).
§ Classified as >50%. Reported as a percentage of those with reported differential counts.
¶ Microbiological diagnoses are composite categories combining microscopy and culture. Cryptococcal meningitis includes positive cryptococcal cultures plus positive cryptococcal microscopy and cryptococcal antigen tests. Tuberculous (TB) meningitis includes positive TB cultures and positive TB microscopy. Pneumococcal meningitis includes positive pneumococcal cultures and positive pneumococcal microscopy. No additional serological or molecular testing was performed routinely, thus further microbiological classification is not possible. Note that the number of cryptococcal meningitis, tuberculous meningitis, and bacterial meningitis exceed the total number of patients with a microbiological diagnosis as there were 45 patients (0.8% of those with a microbiological diagnosis) with confirmed co-infections: 2 with cryptococcal and TB co-infection, 1 with concurrent TB and bacterial meningitis, and 42 with concurrent cryptococcal meningitis and bacterial meningitis.
** Other identified pathogens included 159 gram negative rods (on Gram or culture, primarily *E.coli* and *Klebsiella*), 22 Group B *Streptococcus,* and 21 *S.aureus,* plus 93 other culture or gram positive samples.

Supplementary Table 3. Risk factors for ten-week mortality in pneumococcal meningitis in full cohort and known HIV-infected cohort.

Table S3a. Full cohort univariable

Variable	Category	Data	No	Mortality 10 weeks	HR	p-value
Age	<50 years<br>≥50 years	236 (99·2%)	207 (87·7%)<br>29 (12·3%)	93 (44·9%)<br>19 (65·5%)	Ref<br>1·57 (0·96-2·58)	0·07
Sex	Female<br>Male	238 (100%)	141 (59·2%)<br>97 (40·8%)	65 (46·1%)<br>47 (48·5%)	Ref<br>1·12 (0·77-1·63)	0·56
WCC	100/µL increase	221 (92·9%)	---	---	0·96 (0·92-0·99)	0·009
CSF protein	1 g/L increase	64 (26·9%)	---	---	1·04 (1·00-1·09)	0·046
CSF glucose	1 mmol/L increase	103 (43·3%)	---	---	0·61 (0·35-1·07)	0·09
HIV infected	No<br>Yes	123 (51·7%)	44 (35·8%)<br>79 (64·2%)	36 (81·8%)<br>37 (46·8%)	Ref<br>0·40 (0·25-0·63)	<0·001
Facility level	Primary/district<br>Referral	238 (100%)	64 (26·9%)<br>174 (73·1%)	31 (48·4%)<br>81 (46·6%)	Ref<br>0·97 (0·64-1·46)	0·88

Table S3b. Known HIV-restricted cohort univariable

Variable	Category	Data	No·	Mortality 10 weeks	HR	p-value
Age	<50 years<br>≥50 years	79 (100%)	65 (82·3%)<br>14 (17·7%)	28 (43·1%)<br>9 (64·3%)	Ref<br>1·66 (0·78-3·52)	0·19
Sex	Female<br>Male	79 (100%)	54 (68·3%)<br>25 (31·7%)	27 (50·0%)<br>10 (40·0%)	0·75 (0·36-1·55)	0·44
WCC	100/µL increase	79 (100%)	---	---	0·93 (0·87-0·99)	0·03
CSF protein	1 g/L increase	21 (26·6%)	---	---	0·92 (0·72-1·17)	0·50
CSF glucose	1 mmol/L increase	33 (41·8%)	---	---	0·08 (0·00-11·99)	0·32
Facility level	Primary/district<br>Referral	79 (100%)	29 (36·7%)<br>50 (63·3%)	16 (55·2%)<br>21 (42·0%)	Ref<br>0·67 (0·35-1·29)	0·23

CSF = cerebrospinal fluid; HR = hazards ratio; WCC = white cell count

Supplementary Figure 1. Cerebrospinal fluid (CSF) white cell count (WCC) distribution in culture-negative cases with CSF WCC >20 cells/μL.

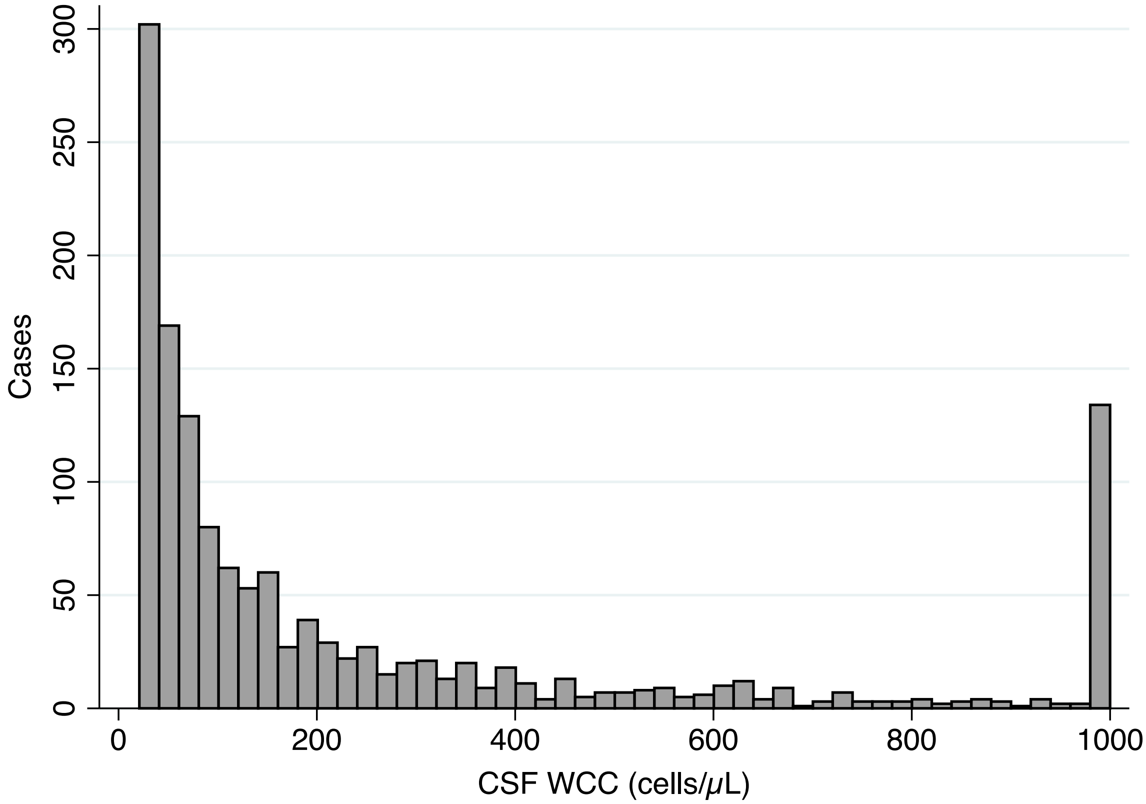

Note: all CSF WCC ≥1000 cells/μL recoded as WCC of 1000 cells/μL
